# Oral 8-aminoguanine against age-related retinal degeneration

**DOI:** 10.21203/rs.3.rs-4022389/v1

**Published:** 2024-05-06

**Authors:** Yuanyuan Chen, Abhishek Vats, Yibo Xi, Amanda Wolf-Johnston, Owen Clinger, Riley Arbuckle, Chase Dermond, Jonathan Li, Donna Stolze, José-Alain Sahel, Edwin Jackson, Lori Birder

**Affiliations:** University of Pittsburgh; University of Pittsburgh; University of Pittsburgh; University of Pittsburgh; University of Pittsburgh; University of Pittsburgh; University of Pittsburgh; University of Pittsburgh; University of Pittsburgh; University of Pittsburgh School of Medicine; University of Pittsburgh; University of Pittsburgh

## Abstract

Visual decline in the elderly is often attributed to retinal aging, which predisposes the tissue to pathologies such as age-related macular degeneration. Currently, effective oral pharmacological interventions for retinal degeneration are limited. We present a novel oral intervention, 8-aminoguanine (8-AG), targeting age-related retinal degeneration, utilizing the aged Fischer 344 rat model. A low-dose 8-AG regimen (5 mg/kg body weight) via drinking water, beginning at 22 months for 8 weeks, demonstrated significant retinal preservation. This was evidenced by increased retinal thickness, improved photoreceptor integrity, and enhanced electroretinogram responses. 8-AG effectively reduced apoptosis, oxidative damage, and microglial/macrophage activation associated with aging retinae. Age-induced alterations in the retinal purine metabolome, characterized by elevated levels of inosine, hypoxanthine, and xanthine, were partially mitigated by 8-AG. Transcriptomics highlighted 8-AG’s anti-inflammatory effects on innate and adaptive immune responses. Extended treatment to 17 weeks further amplified the retinal protective effects. Moreover, 8-AG showed temporary protective effects in the *Rho*^*P23H/+*^ mouse model of retinitis pigmentosa, reducing active microglia/macrophages. Our study positions 8-AG as a promising oral agent against retinal aging. Coupled with previous findings in diverse disease models, 8-AG emerges as a promising anti-aging compound with the capability to reverse common aging hallmarks.

## Introduction

Aging, an intrinsic, programmed biological process inherent to all living organisms, is characterized by a continuum of morphological and functional transformations within organ systems, which are evident even during healthy aging. Notably, oxidative damage and chronic inflammation stand out as primary factors that either precipitate or exacerbate the aging process (1). The quest for a treatment to prevent or reverse aging has been a constant endeavor that can be traced back to the earliest civilizations (2). With the prolongation of the human lifespan in recent decades, the demand for medical interventions addressing age-associated disorders has increased significantly. Targeting the molecular underpinnings of aging and developing integrative therapies to decelerate or reverse the aging process across various organ systems are emerging as promising approaches to combat age-related diseases.

Declined vision is a common health issue among the elderly. Beyond the optic changes of the aging eye, the neural retina grows thinner with age. It has been reported that rods in the central retinal area decrease by 30% from the third to the ninth decade of life in humans (3, 4), a change accompanied by a functional decline in rod performance as evidenced by a more rapid decline of peripheral visual sensitivity, delayed dark adaptation and decreased electroretinogram (ERG) a- and b-waves. Age-related loss of retinal ganglion cells (RGCs) is proportional to the loss of rods, leading to a constant rod:RGC ratio with aging. Interestingly, cone densities in the fovea — which are critical for central vision and visual acuity — remain unchanged from the ages between the twenties and nineties. However, in the pathological context of age-related macular degeneration (AMD), pericentral rods followed by foveal cones are predominantly affected (5, 6). AMD is a complex condition influenced by both environmental and genetic factors, with age being the primary non-modifiable risk factor that significantly influences disease progression. The incidence of AMD quadruples with each passing decade after 55 years of age (7). Hence, the age-related changes in the normal retina must be considered to understand the role of aging in AMD. In addition to the progressive loss of rods and RGCs, age-related retinal changes also include alterations of Bruch’s membrane (8, 9), accumulation of autofluorescent and electron-dense lipofuscin granules in the retinal pigment epithelium (RPE) (10–12), and the chronic elevation of adaptive and innate inflammatory processes, called para-inflammation (13, 14). In response to age-related oxidative and metabolic cell stress, characterized by the activation of residential microglia and myeloid-derived macrophages as well as the complement pathway, dysregulation of para-inflammation may precipitate pathological conditions.

Purine metabolism is crucial for the well-being of terminally differentiated neurons as purine metabolites are carriers of energy (ATP and GTP), building blocks for DNA and RNA biosynthesis, secondary messengers (cAMP and cGMP), coenzymes (NAD and NADP), and signaling molecules for purine receptors. In mammals, purine nucleotides are generated mainly via the *de novo* purine biosynthesis pathway and the purine salvage pathway (15), the latter being particularly significant for the brain and heart which require rapid regeneration of these molecules due to their high energy demands.

**Figure 1:**
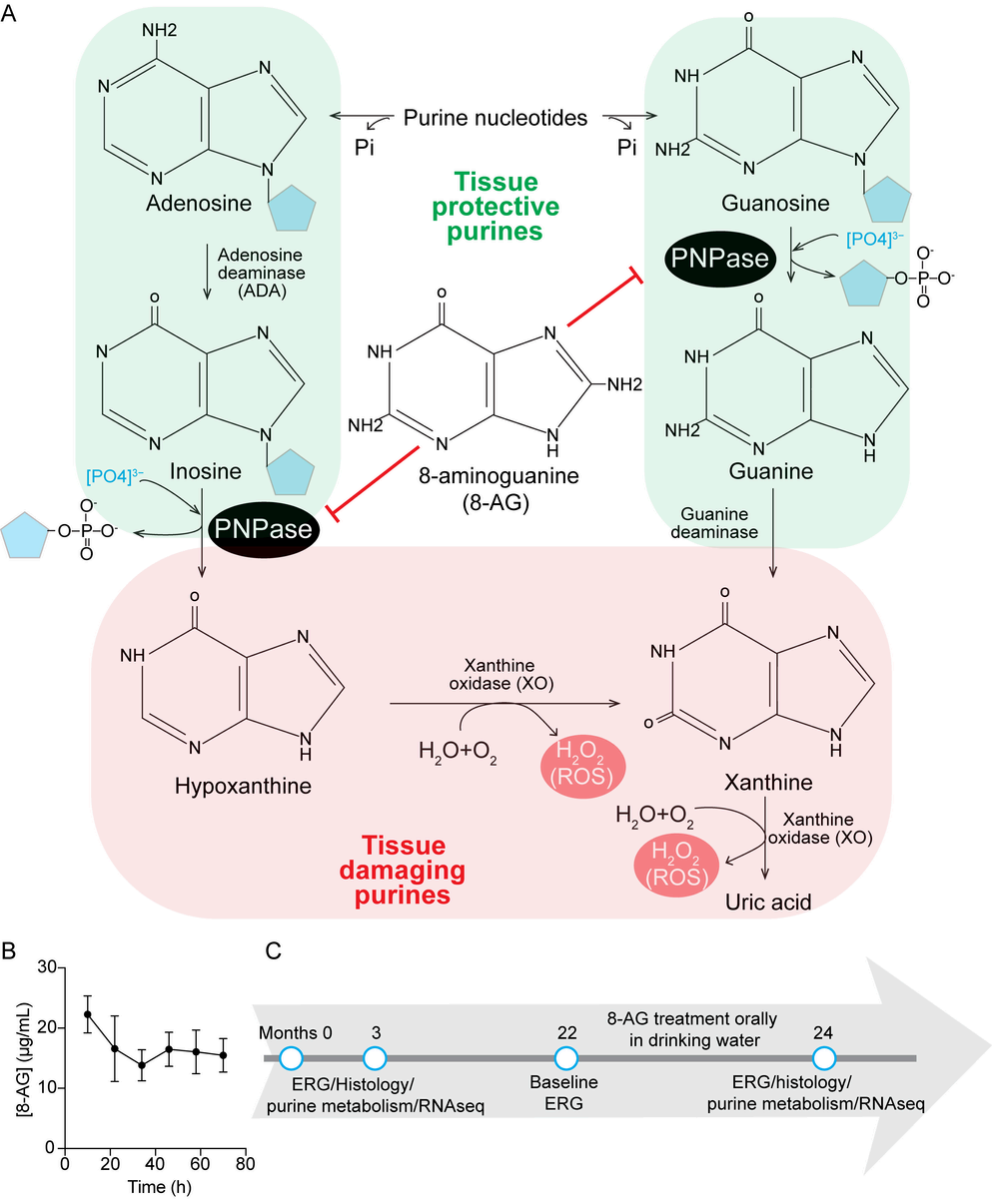
Purine metabolism and treatment regimen for rats. **A.** A diagram shows the catabolism of the purine nucleosides, adenosine and guanosine. The tissue-protecting purines are shown in a light green background and tissue-damaging purines are in a light orange background. Black oval shows the activity of PNPase and 8-aminoguanine (8-AG) in the center shows its affected reaction cascade. The light blue pentagon is ribose sugar. ROS; reactive oxygen species. **B.** Stability of 8-AG in water at room temperature for 3 days using HPLC, showing only slight decay of 8-AG from 22 to 17 μg/mL in 24 hour. **C.** Treatment regimen of the experiments, performed on rats. Young untreated rats were 3 months old, aged untreated rats were 22 months old when started on oral 8-AG treatment for 8 weeks. The baseline data for OCT and ERG were collected before 8-AG treatment at 22 months.

The purine salvage pathway mediates recycling of inosine and guanosine and requires purine nucleoside phosphorylase (PNPase) to convert inosine to hypoxanthine and guanosine to guanine, which in turn are substrates for hypoxanthine-guanine phosphoribosyltransferase (HGPRT) to regenerate purine nucleotides (16). An alternative to the HGPTR pathway is the conversion of guanine to xanthine by guanine deaminase and the oxidation of hypoxanthine to xanthine by xanthine oxidase (XO); both pathways lead to uric acid formation via XO-mediated oxidation of xanthine (Fig. 1A). Because XO-catalyzed reactions generate reactive oxygen species, for example, H_2_O_2_, PNPase-mediated accumulation of hypoxanthine and xanthine may promote oxidative tissue damage. In addition, PNPase activity reduces inosine and guanosine levels, thus altering the balance between inosine-guanosine (which exert antioxidant, anti-inflammatory and tissue-protective effects (17–23) versus hypoxanthine-xanthine (which promote oxidative stress). This balance may be a key determinant of postmitotic retinal health. Notably, such an imbalance in purine metabolomic balance is evident in brain injuries such as stroke, characterized by depleted ATP and accumulated xanthine and uric acid (15, 24). Nonetheless, the significance of changes in retinal purine levels during aging remains poorly understood.

Emerging evidence suggests that rebalancing the purine metabolome by inhibiting PNPase provides health benefits and tissue-protective effects in multiple organ systems and diseases. Recently, Jackson et al. discovered that in rats 8-aminoguanine (8-AG), an endogenous PNPase inhibitor (25), promotes diuresis, natriuresis and glucose excretion and attenuates salt-induced hypertension (26, 27). These beneficial renal and cardiovascular effects of 8-AG are due at least in part to the inhibition of PNPase (28), leading to elevated inosine levels in the kidney (29), which in turn activate adenosine A_2B_ receptors (29), thereby increasing renal medullary blood flow (29). This increase may mediate, in part, the effects of 8-AG on renal excretion of sodium, glucose and water, and may mediate, in part, the antihypertensive effects of 8-AG. As recently reviewed (30), Jackson and coworkers also showed that oral administration of 8-AG, and/or its prodrug 8-aminoguanosine (27), prevents strokes and extends lifespan in Dahl SS rats on a high-salt diet, attenuates progression of pulmonary hypertension and improves outcomes in animal models of the metabolic syndrome and sickle cell disease.

The above findings motivated our recent investigation of the anti-aging/reverse-aging effects of PNPase inhibition in the bladder (31) and urethra (32). In these studies, we observed that 8-AG reverses age-associated bladder and urethral abnormalities in aged Fischer 344 (F344) rats. Most notably, in aged F344 rats 8-AG effectively restored the lower urinary tract to a youthful and healthier cellular, structural, and functional state (31, 32).

The F344 rat is a widely used model for studying aging-related effects across various organ systems. Age-related abnormalities in this strain include reduced locomotor activity (33), progressive hearing loss (34), cognitive decline (35), nephropathy, fibrous tissue accumulation in the heart (36), impaired lower urinary tract function (37, 38), and retinal degeneration (39–42). Thus, we considered investigating further the anti-aging/reverse-aging effects of 8-AG in other age-associated diseases, with a focus on retinal degeneration.

F344 rats experience progressive age-related retinal thinning and light-induced retinal degeneration (39–42). Photoreceptor loss starts at the peripheral ends at 12–18 months of age, and as the animal ages the thinning of the outer nuclear layer (ONL) extends to the central retinae. Similarly, there is a reduction in inner retinal neurons, including those in the inner nuclear layer (INL) and RGC layer. In addition, aging also causes axon loss in the optic nerve of these rats (42). While the rodent model is not ideal for studying AMD due to the absence of a cone-enriched area in their retinae, the F344 rat provides a valuable model for studying age-associated retinal neuron loss and assessing potential therapies aimed at reversing age-related retinal degeneration. Here, we present our findings that inhibiting PNPase with 8-AG ameliorates oxidative damage, reduces retinal immune responses and slows the progression of retinal degeneration in this rat model of retinal aging.

## Results

### Treatment regimen.

To assess 8-AG’s effect on age-related retinal degeneration, we administered 5 mg/kg/day of 8-AG to 22-month-old F344 rats via drinking water over 8 weeks. We observed that 8-AG remains stable in water at room temperature for up to 3 days (Fig. 1B), allowing for consistent daily dosing through daily water replacement with fresh 8-AG solution. We evaluated the efficacy, safety, and mechanism of action of 8-AG through ERG and endpoint assessments as illustrated in Fig. 1C.

### 8-AG improves the function of aged rat retinae.

**Figure 2:**
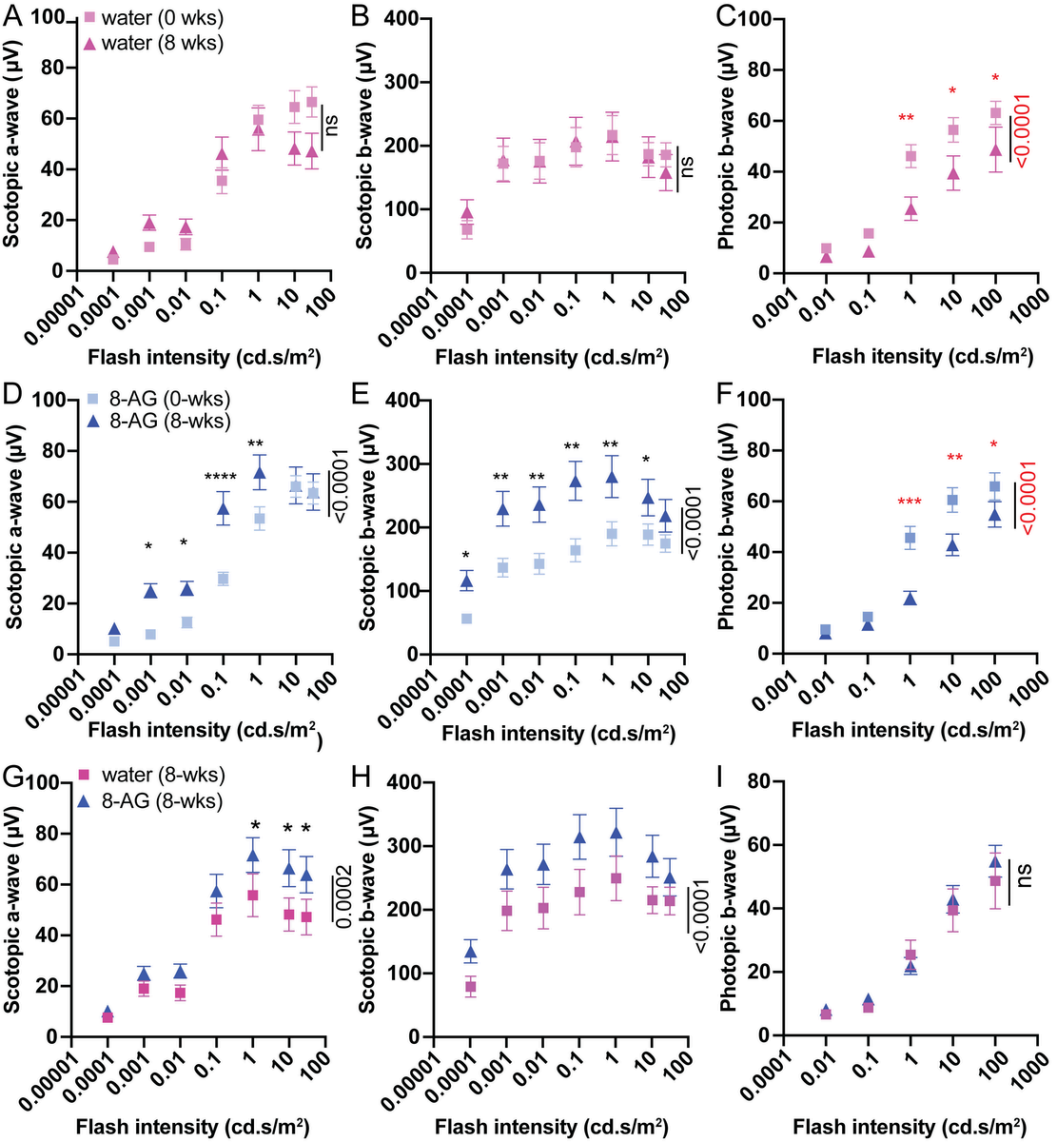
Functional protection of retinae in aged Fisher 344 rats by 8-AG. Twenty-two-month-old aged Fischer 344 rats were supplemented with 8-AG in drinking water at 5 mg/kg for 8 weeks. Electroretinograms (ERG) were recorded for baseline at 0 weeks (at 22 months), and after 8 weeks of treatment (at 24 months). Aged Fischer 344 rats not treated with 8-AG were included as controls. **A-I** are the semi-log plots of ERG wave form responses in μV as a function of flash intensities (cd.s/m^2^) for water and 8-AG treated F344 rats. **A, B,** and **C** are the scotopic a-wave, scotopic b-wave, and photopic b-wave responses, respectively for the control rats treated with only water at 0 weeks (light magenta squares, baseline) and 8 weeks (dark magenta triangles). **D, E,** and **F** are the scotopic a-wave, scotopic b-wave, and photopic b-wave responses, respectively for 8-AG treated rats at 0 weeks (light blue squares, baseline) and after 8 weeks of 8-AG treatment (dark blue triangle). **G, H,** and **I** are the scotopic a-wave, scotopic b-wave, and photopic b-wave responses, respectively after 8 weeks (at 24 months) of treatment with 8-AG (dark blue triangle) and water only (dark magenta triangle). N=16, combining two independent sets of experiments. Statistical analysis was done using two-way ANOVA, *, **, ***, ****, *P*<0.05, 0.01, 0.001, 0.0001, respectively, ns is non-significant. Data points and error bars are means and SEMs, respectively.

We recorded scotopic and photopic ERGs at baseline and after 8 weeks of treatment in the aged rats. Aged rats showed substantially lower scotopic and photopic ERG responses than young ones, indicating diminished rod and cone functions (Supplementary Figure S1). From 22 to 24 months of age, control rats showed stable scotopic a- and b-waves (Fig. 2A&B) but decreased photopic responses (Fig. 2C). In contrast, 8-AG treated animals showed a left-shift of scotopic a-wave response curve (*P* < 0.0001, Fig. 2D) and increased scotopic b-wave responses (*P* < 0.0001, Fig. 2E). Scotopic a- and b-wave responses to 0.1 cd.s/m^2^ flashes increased from 30 and 164 μV to 57 and 274 μV, respectively (*P* < 0.0001 and < 0.01, Fig. 2D&E), indicating improved rod photosensitivity and function during the treatment period. Photopic b-wave responses of the 8-AG treated animals decreased similarly to controls (P < 0.001, Fig. 2C&F). After 8 weeks, 8-AG treated animals had significantly higher scotopic a- and b-wave responses than the water-treated control group (*P* = 0.0002 and < 0.0001; Fig. 2G and 2H, respectively), but similar photopic responses (Fig. 2I). Thus, 8-AG treatment for eight weeks enhanced, rather than merely preserved, the rod functions in aged F344 rats, without notably affecting cones.

### 8-AG improves the structure of aged rat retinae.

**Figure 3:**
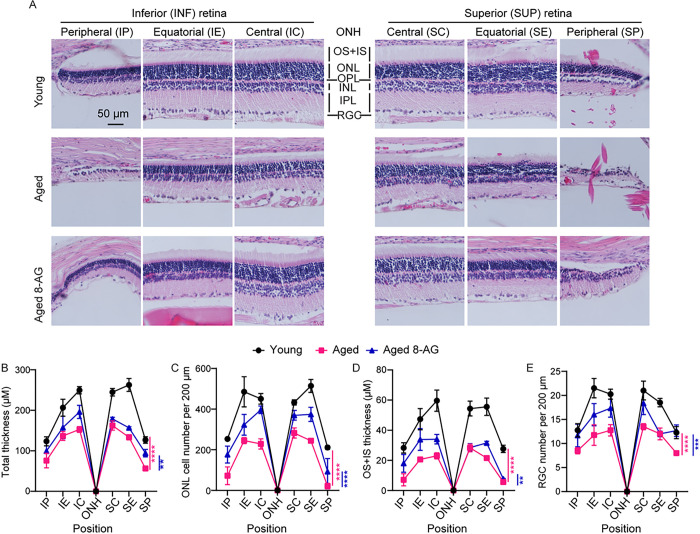
Retinal hematoxylin and eosin (H&E) staining shows potent retinal protection by 8-AG in aged Fischer 344 rats. The experimental Fischer 344 rats were untreated young (3 months), untreated aged (24 months), or aged treated with 8-AG at 5 mg/kg bw daily in drinking water for 8 weeks starting at 22 months of age. Rats were euthanized and eyes were enucleated and prepared for paraffin-embedded H&E staining. **A.** Representative H&E-stained images on the superior (S) and inferior (I) sides of optic nerve head (ONH) at central (SC&IC), equatorial (SE&IE) and peripheral (SP&IP) regions of retinal paraffin sections. Scale bar, 50 μm. OS+IS, outer and inner segments; ONL, outer nuclear layer, OPL, outer plexiform layer; INL, inner nuclear layer; IPL, inner plexiform layer; RGC, retinal ganglion cells. **B-E.** Spidergrams of measurements from H&E staining images as shown in **A,** including total thickness of all retinal layers (**B**), cell number in ONL per 200 μm length of the retinal section (**C**), thickness of OS+IS (**D**), number of cells in retina ganglion cells layer (RGC) per 200 μm length of the retinal section (**E**). N=8. Data points and error bars are means and SEMs, respectively, **, ***, ****, p<0.01, 0.001, 0.0001, respectively, analyzed by two-way ANOVA.

After an 8-week 8-AG regimen, we conducted histological analysis on the retinae (Figs. 3A and S2). The untreated aged rats, as compared to young rats, showed notable age-related thinning, reduced nuclei in the ONL, diminished outer and inner segment (OS + IS) layers, and fewer nuclei in RGC layer (Fig. 3A–E). The most severe degeneration occurred in the superior peripheral region, with a complete loss of photoreceptor layers in some of the aged retinae (Fig. 3A–D). Conversely, the 8-AG treated animals showed significantly thicker retinae (*P* < 0.01), higher ONL cell count (*P* < 0.0001), thicker OS + IS layer (*P* < 0.001), and increased cells count in the RGC layer (*P* < 0.001), compared to untreated controls (Fig. 3A–E). Particularly, 8-AG preserved the ONL at the peripheral ends, which was absent in some control retinae (Fig. 3A). The histological data indicate that 8 weeks of oral 8-AG initiated at 22 months of age provides highly effective protection against age-related retinal degeneration in F344 rats.

### 8-AG reduces cell death and apoptosis in aged rat retinae.

**Figure 4:**
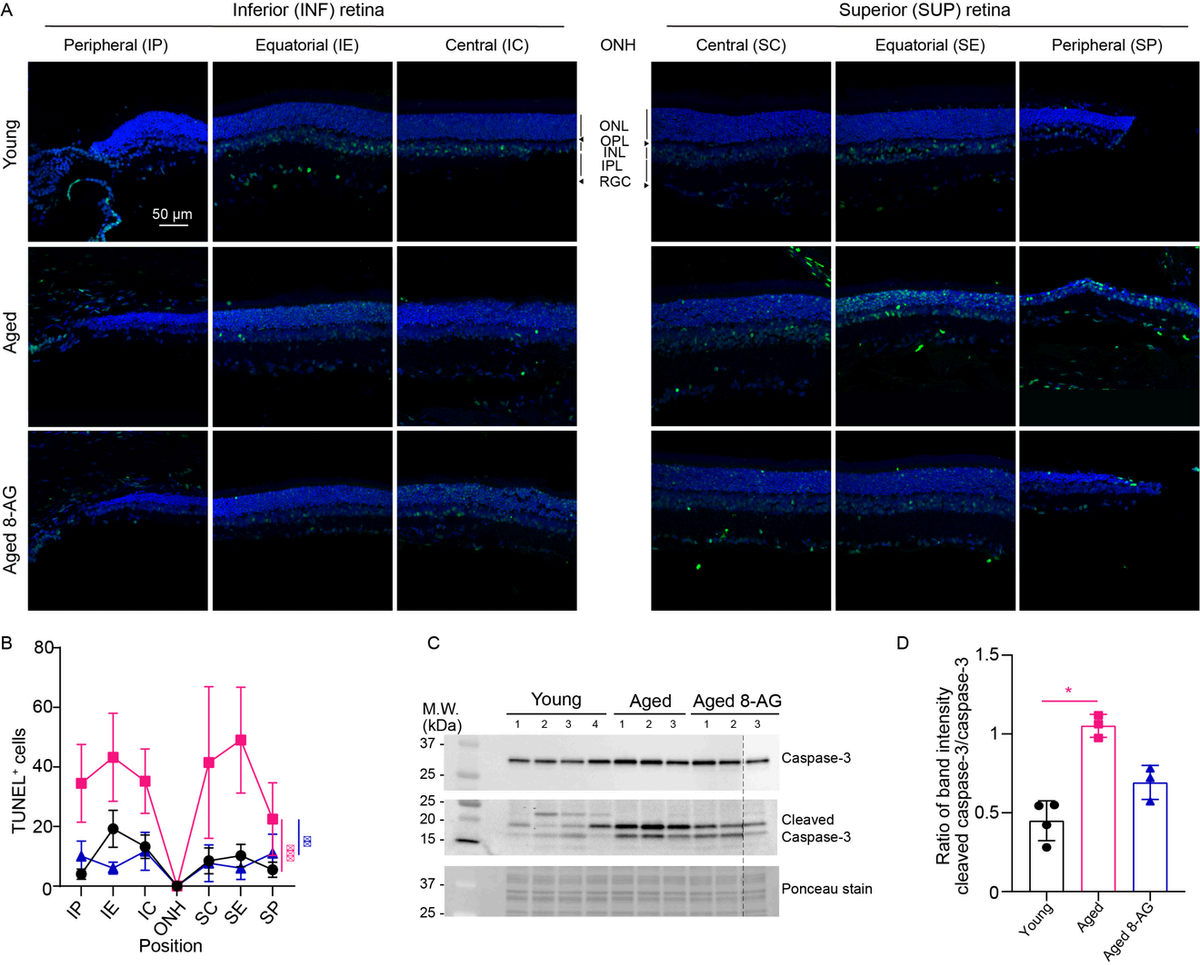
8-AG is safe and reduces apoptosis in aged Fischer 344 rats. The experimental Fischer 344 rats were untreated young (3 months), aged (24 months), or aged treated with 8-AG at 5 mg/kg bw daily in drinking water for 8 weeks starting at 22 months of age. Rats were euthanized and eyes were enucleated and prepared for cryo-sectioning and TUNEL staining for quantification of dead cells in the retinae. **A.** TUNEL stained retinal cryosections on the superior (S) and inferior (I) sides of optic nerve head (ONH) at central (SC&IC), equatorial (SE&IE) and peripheral (SP&IP) regions of retinal cryosections. Scale bar, 50 μm. Green, TUNEL positive (TUNEL^+^) cells, and blue, Hoechst 33342 staining for nucleus. **B.** The Spidergrams of TUNEL^+^ cell numbers at different positions of the retinae. Black circles, young rats; magenta squares, aged rats; blue triangles, aged rats treated with 8-AG. N=3–4. Data points and error bars are means and SEMs, respectively, ** and ***, p<0.01, 0.001, respectively, analyzed by two-way ANOVA **C.** Immunoblots against the intact (top panel, 30 kDa) and cleaved caspase-3 (middle panel, 17 and 19 kDs) from rat retinae. Ponceau stain (bottom panel) was used as the loading control. Each number represents one rat retina. All samples are from the same membrane, dotted line represents the joined membrane. **D.** Column plot of the band intensity ratio of cleaved to intact caspase-3. N=3–4. Columns and error bars are means and SDs, respectively. *, *p*<0.05 analyzed by Kruskal Wallis.

We assessed retinal cell death by the terminal deoxynucleotidyl transferase dUTP nick end labeling (TUNEL assay) (Figs. 4A&B and S3) and caspase-3 cleavage via immunoblotting (Figs. 4C&D and S4). Even healthy young retinae had some TUNEL^+^ cells in the INL and RGC layer. Aged retinae had over double the TUNEL^+^ cells compared to young ones (P < 0.001), but 8-AG treatment reduced TUNEL^+^ cells to levels close to young retinae (Fig. 4A&B, P < 0.01 comparing 8-AG vs. aged). Caspase-3 cleavage, a key apoptotic event, initiates a caspase cascade leading to programmed cell death (43). Immunoblotting revealed aging doubled the cleaved-to-intact caspase-3 ratio; 8-AG treatment reduced this ratio by 1.5-fold (Figs. 4C&D and S4), indicating it lowers age-related retinal apoptosis. These data indicates that oral 8-AG is safe and decreases cell death in aged retinae.

### 8-AG rescues rhodopsin level in rod photoreceptors and preserves cones.

**Figure 5:**
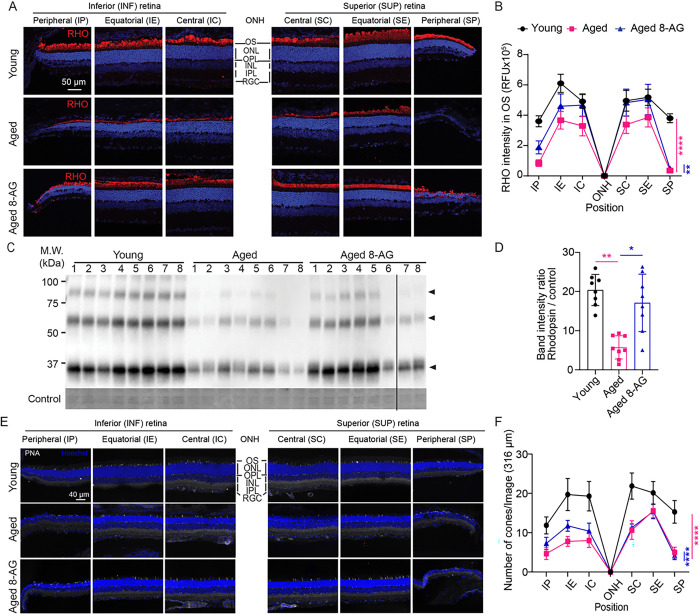
8-AG protects photoreceptors. The experimental Fischer 344 rats were untreated young (3 months), aged (24 months), or aged treated with 8-AG at 5 mg/kg bw daily in drinking water for 8 weeks starting at 22 months of age. Rats were euthanized at the end of treatment and eyes were enucleated and prepared for cryo-sectioning and immunostaining against rhodopsin (RHO) and peanut agglutinin (PNA) staining for cones. **A.** Immunofluorescence images of RHO (red) and Hoechst 33342 for nucleus (blue) on the superior (S) and inferior (I) sides of optic nerve head (ONH) at the central (SC&IC), equatorial (SE&IE) and peripheral (SP&IP) regions of retinal cryosections. Scale bar, 50 μm. **B.** Spidergram of RHO immunofluorescence intensity in the OS at different positions of the retinae. **C.** Immunoblots of RHO in rat retinae. Each number represents one rat retina. Arrowheads show tetramer, dimer, and monomer of RHO from top to bottom, respectively. **D.** Bar graph of RHO immunoblot intensity normalized by loading control. **E.** Immunofluorescence images of PNA and Hoechst 33342 for nucleus (blue) on the superior (S) and inferior (I) sides of optic nerve head (ONH) at SC&IC, SE&IE, and SP&IP regions of retinal cryosections. Scale bar, 40 μm. **F.** Spidergram of number of PNA positive cones at different positions of the retinae. Black circles, young rats; magenta squares, aged rats; blue triangles, aged rats treated with 8-AG. For spidergram, data points and error bars are means and SEMs. N=8. **, ****, P<0.05 and 0.0001 by two-way ANOVA. For bar graph, column and error bars are means and SD. N=8, *, **, P<0.05 and 0.01, respectively, by Kruskal Wallis.

As we observed that 8-AG increased the rod function (Fig. 2D,E,G,and H) and rescued the length of OS + IS layers (Figs. 3A&D and S2) in the aged F344 rat retinae, we then investigated if 8-AG affects the level of rhodopsin (RHO), the most abundant phototransduction components in rods ( Figs. 5, S5 and S6). RHO levels decreased considerably in the aged retinae compared to young controls, shown by both immunohistochemistry (IHC) (Fig. 5A&B, *P* < 0.0001) and immunoblots (Fig. 5C&D, over 3-fold decrease, *P* < 0.01). 8-AG treatment markedly increased RHO levels, over 2-fold compared to the control (*P* < 0.01 and < 0.05 in Fig. 5B and D, respectively). However, 8-AG treatment did not significantly increase other phototransduction components, such as ARRESTIN1 and PDE6B, in the aged rat retinae (Supplementary Figures S7 and S8). These findings suggest that 8-AG reverts the rods from a senescence-like state to a healthier and functional state, characterized by the restored OS + IS morphology, RHO levels, and rod function.

While 8-AG didn’t improve cone function within the 8 weeks treatment period (Fig. 2), a significant higher number of cones in the inferior retinae of 8-AG-treated aged rats were observed (Figs. 5E&F and S9), indicating some degree of cone protection.

### Oral 8-AG reduces oxidative damage in Fischer 344 rats.

**Figure 6:**
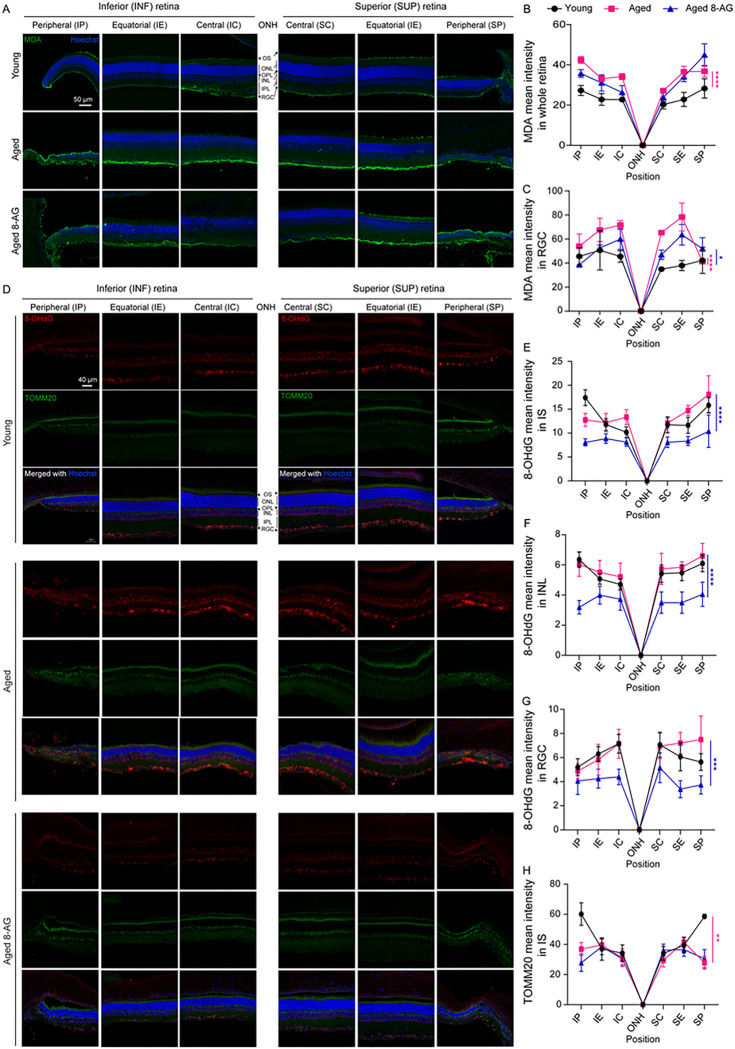
8-AG treatment reduces oxidated DNA damage in Fischer 344 rats. Aged Fischer 344 rats were treated with 8-AG at 5 mg/kg body weight (bw) daily for 8 weeks, starting at 22 months of age. Aged (24 months) and young (3 months of age) untreated rats received normal water. Rats were euthanized at the end of treatment and eyes were isolated and retinal cryosection were made for immunostaining against malondialdehyde (MDA) as an indicator of lipid peroxidation, 8-hydroxy-2’ -deoxyguanosine (8-OHdG) as a marker for oxidated DNA, and Translocase of Outer Mitochondrial Membrane 20 (TOMM20) as a mitochondrial marker. **A.** Immunofluorescence images of MDA imaged at six positions of the retinae from young, aged untreated, and aged 8-AG treated rats. ONH, optic nerve head; INF, inferior; Sup, superior; C, central; E, equatorial; P, peripheral. Green, MDA; and blue, Hoechst 33342 for nuclear staining. **B** and **C.** Spidergrams of MDA intensity in the whole retina and in the retinal ganglion cell layer (RGC), respectively. Data points represent the central, equatorial, and peripheral of INF and SUP parts of retina. **D.** Immunofluorescence images of the 8-OHdG (red), TOMM20 (green), and merged channel with Hoechst 33342 (blue) on retinal cryosections of young, aged and 8-AG treated aged rats. **E-G.** Spidergrams of immunofluorescence intensity of 8-OHdG on the inner segment layer (IS) (**E**), inner nuclear layer (INL) (**F**), and RGC layer (**G**). **H.** Spidergram of immunofluorescence intensity of TOMM20 on IS, where mitochondria reside in photoreceptors. N=3 for MDA staining, N=4–5 for 8-OHdG and TOMM20. Data points and error bars are means+SEM. * and ****, *P*<0.05 and 0.0001, respectively, analyzed by two-way ANOVA.

To investigate if 8-AG reverses aging-associated oxidative damage, we used IHC to assess malondialdehyde (MDA) level for lipid oxidation, 8-hydroxy-2’-deoxyguanosine (8-OHdG) for DNA oxidation (44, 45), and a mitochondria marker, translocase of outer mitochondrial membrane 20 (TOMM20), in young (3 m), aged (24 m) and 8-AG treated aged Fischer 344 rat retinae (Fig. 6). Aged retinae exhibited increased MDA level, especially in the RGC layer (Figs. 6A–C, S10, P < 0.0001). 8-AG did not alter overall retinal MDA levels but significantly decreased MDA staining in the RGC layer (Figs. 6A–C, S10, P < 0.05), suggesting 8-AG protects RGCs specifically from lipid peroxidation. No obvious difference was observed in MDA staining in photoreceptor OS, ONL, and INL layers. Perhaps, the potential reason that the lipid-enriched photoreceptors OS layer is more resistant to lipid oxidation may be due to its continuous renewal and phagocytosis of OS tips by the RPE cells.

Moreover, while we noted marginal increases in 8-OHdG in aged retinae, implying minimal DNA oxidative damage, 8-AG significantly reduced 8-OHdG in photoreceptor IS, INL, and RGC layers (Figs. 6D–G and S11), indicating mitigated DNA oxidation. No significant change in TOMM20 was observed with 8-AG treatment (Figs. 6D&H and S11). Thus, 8-AG exhibits potent antioxidant effects in the neural retina, reducing RGC lipid oxidation and DNA oxidation in photoreceptor mitochondria and nuclei of inner retinal neurons. We noticed that the ONL is free of 8-OHdG staining, suggesting little oxidative damage to the chromosomal DNA of photoreceptor cells, potentially due to the condensed form of chromatin.

### 8-AG treatment reduces the number of injury-induced Müller glia and microglia in rat retina.

**Figure 7:**
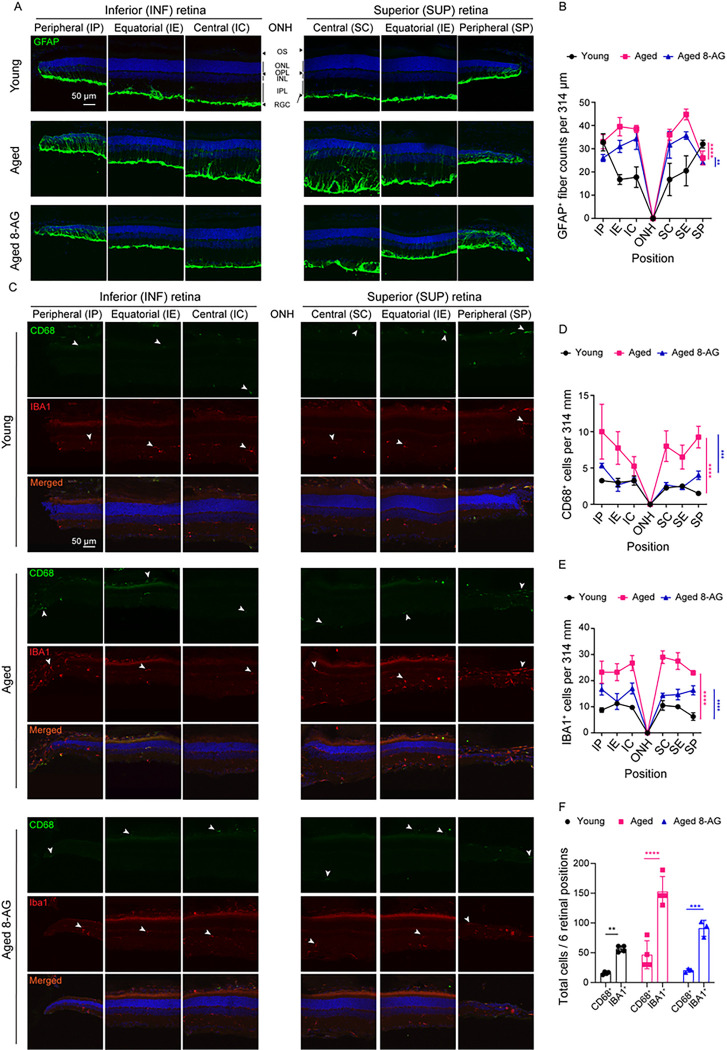
8-AG reduces the number of activated Müller glia and microglia/macrophages in aged Fischer 344 rat retinae. The experimental Fischer 344 rats were untreated young (3 months), aged (24 months), or aged treated with 8-AG at 5 mg/kg bw daily in drinking water for 8 weeks starting at 22 months of age. Rats were euthanized and eyes were enucleated and prepared for cryo-sectioning and immunostaining against glial fibrillary acidic protein (GFAP) as marker of activated Müller glia, the cluster of differentiation 68 (CD68) and ionized calcium-binding adaptor molecule 1 (IBA1) as markers of microglia/macrophages. Hoechst 33342 were used to stain the nucleus in blue. **A** and **C** are representative immunostaining images of GFAP (green in A), CD68 (green in C) and IBA1 (red in C) retinal cryosections on the superior (S) and inferior (I) sides of optic nerve head (ONH) at central (SC&IC), equatorial (SE&IE) and peripheral (SP&IP) regions of the retina. Scale bar, 50 μm. The white arrowheads in (C) indicate the CD68^+^ or IBA1^+^ cells in the retinae. **B, D,** and **E** are spidergrams of the numbers of GFAP^+^ fibers (B), CD68^+^ cells (D), and IBA1^+^ cells (E), respectively, at different retina positions, calculated from immunostainings shown in **A** and **C. F.** Bar graph represent the total CD68^+^ and IBA1^+^ cells in individual retinal at six positions. Black circles, young rats; magenta squares, aged rats; blue triangles, aged rats treated with 8-AG. N=3–4. Data points and error bars are means and SEMs in B, D, and E and means and SDs in F. **, ***, ****, *p*<0.01, 0.001 and 0.0001, respectively, analyzed by two-way ANOVA in B, D, E, and by Kruskal Wallis in F.

Glial cells play a supportive role in maintaining the structural and functional stability of the central nervous system (CNS) (46). Müller glia and microglia are activated in response to retinal neuron stress. We immunostained F344 rat retinae for activated Müller glia with glial fibrillary acidic protein (GFAP, Figs. 7A&B, S12) and for phagocytic macrophage/microglia with cluster of differentiation 68 (CD68) and ionized calcium binding adaptor molecule 1 (IBA1, Figs. 7C–F, S13, and S14). Aged retinae had increased number of GFAP^+^ filaments throughout, which 8-AG reduced in central and equatorial regions (Figures. 7A&B, S12, P < 0.01), but not in severely degenerated peripheries. Similarly, 8-AG reduced CD68^+^ and IBA1^+^ cell numbers that were elevated in the untreated aged rat retinae, indicating its anti-inflammatory effects (Figures. 7C–F, S13 and S14, P < 0.0001). Notably, all CD68^+^ cells were IBA1^+^, but not vice versa, suggesting distinct macrophage subpopulations. Collectively, we show that age-related increases of activated Müller glial filaments and macrophage/microglia were effectively reduced by 8-AG treatment, suggesting its potential in mitigating age-related retinal inflammation.

### Age-related accumulation of autophagosomes is reduced by 8-AG treatment.

**Figure 8:**
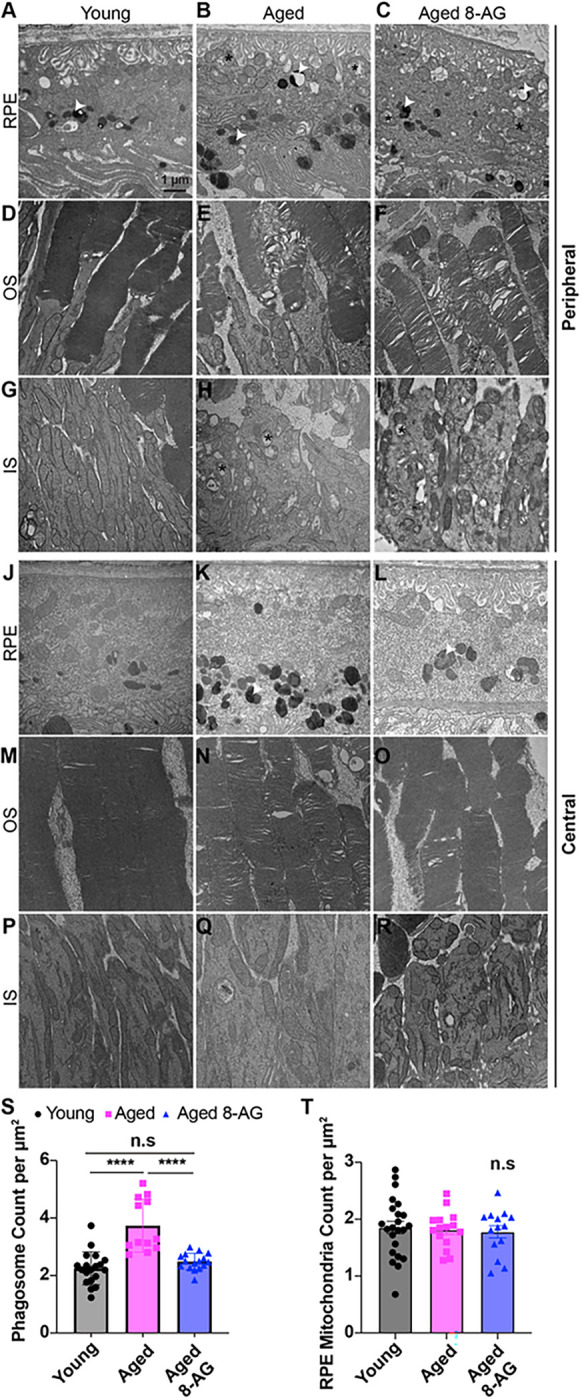
8-AG reduces the number of phagosomes in aged Fischer 344 rat retinae. Young (3 months), aged (24 months), or aged Fischer 344 rats treated with 8-AG at 5 mg/kg bw daily in drinking water for 8 wks. Rats were euthanized and enucleated eyes were processed for TEM. **A-R.** TEM images of young, aged, and aged 8-AG treated rat’s RPE (A-C and J-L), photoreceptor’s outer segment (OS) disc membrane (D-F and M-O) and inner segment (IS) (G-I and P-R) at peripheral (A-I) and central region (J-R) of the retina. **A-C** and **J-L.** TEM images of RPE from the peripheral and central region, respectively, show the autophagosome (white arrowhead) and mitochondria (black asterisk). **D-F** and **M-O.** TEM images show the integrity of photoreceptors’ OS disc membrane at the peripheral and central retina, respectively. **G-I** and **P-R.** TEM images of mitochondria (black asterisk) in the photoreceptor’s IS of the peripheral and central region of rat’s retina, respectively. **S** and **T.** Bar graph representing the number of phagosomes and mitochondria, respectively, in the RPE at the peripheral region of young (black circle), aged (magenta square), and aged 8-AG treated (blue triangle) rat’s retina. N=6–7. Data points and error bars in images S and T are means and SD, ****, indicate *p*<0.0001, analyzed by Kruskal Wallis.

Transmission electron microscopy (TEM) of retinal cross-sections taken at the central and peripheral areas of the retinae of young, aged and 8-AG-treated aged rats (Fig. 8 and Supplementary Data File 1) revealed more severe structural damage in the peripheral retina of aged rats. These damages include swollen mitochondria (marked with “*”), abundant electron-dense phagosomes (marked with “ ”), disorganized photoreceptor OS membranes, and fragmented inner segment (IS) mitochondria, as compared to the central region (Fig. 8A–R). Notably, IS diameter increased with age (Fig. 8G,H,P,Q), whereas the OS diameter was stable (Fig. 8D,E,M,N). 8-AG significantly reduced the age-related accumulation of the phagosomes in RPE (Fig. 8S), with no effects on the number and morphology of mitochondria in the RPE and IS (Fig. 8T). This result suggests that 8-AG treatment restored RPE phagocytosis flux which was compromised by aging.

### Transcriptome of 8-AG treated retina shows downregulation of immune and stress responses.

**Figure 9:**
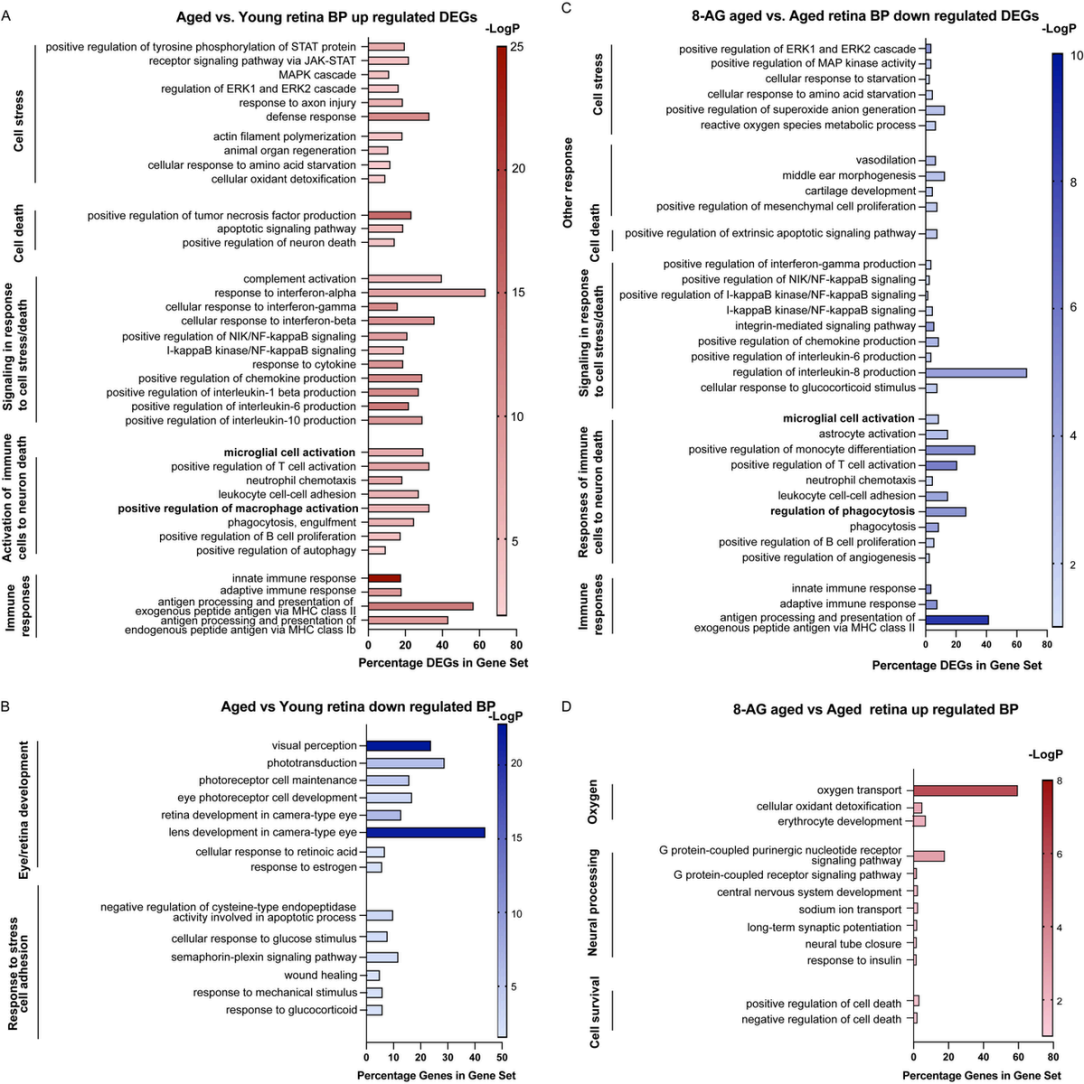
Transcriptomics reveal reduced immune and stress responses in 8-AG treated rats. Young, aged, and 8-AG-treated aged rats’ whole retinae were processed for transcriptomic analysis using bulk RNA-seq. The differentially expressed genes (DEGs) among aged untreated vs young and aged 8-AG treated vs aged untreated were identified which showed >1.5-fold changes with *p*-values of <0.05. Significantly affected biological processes were identified by gene ontology (GO) analysis. **A** and **B.** are the biological pathways (BPs) of up-regulated and down-regulated DEGs, respectively, in the aged untreated vs young rat’s whole retina. **C** and **D.** are the BPs of down-regulated and up-regulated DEGs, respectively, in the aged 8-AG treated vs aged rat’s whole retina. X-axis represents the percentage of genes in a gene set of certain BPs and the Y-axis represents the BPs. On the right-side labeling of all graphs list the cellular pathways.

To explore the molecular changes caused by aging and 8-AG treatment, we isolated retinae and RPE/choroids from young, aged and 8-AG treated aged rats (8 weeks treatment) and performed bulk RNA-Seq (Figure S15, Supplementary Data Files 2–5, and GEO accession number GSE254123). A total of 293 upregulated and 814 downregulated differentially expressed genes (DEGs) were identified, comparing the aged vs young retina, with over 1.5-fold difference and *P* < 0.05 (Supplementary Data File 2). Gene ontology (GO) pathway analysis showed that upregulated genes in aged retinae were associated with stress responses like JAK-STAT, MAPK, and ERK cascades, axon injury response, and defense mechanisms (Fig. 9A). Retina cell remodeling and stress in the aged retina is also reflected by the activation of pathways including actin filament polymerization, response to tumor necrosis factor, cellular response to amino acid starvation, and cellular oxidation detoxification (Fig. 9A). Importantly, aged retinae showed upregulation of genes involved in the pro-inflammatory signaling and production of pro-inflammatory cytokines (Fig. 9A, complement activation, response to interferons-alpha, -beta, and - gamma, NF-kappaB signaling, response to cytokines, positive regulation of chemokines, interleukin-1 beta (IL1b), IL-6 and IL10). Consequentially, biological pathways (BPs) related to the activation of immune cells were observed to be significantly upregulated (Fig. 9A, the activation of microglial cell, T cell, neutrophil, leukocyte, macrophage, B cells and upregulation of phagocytosis and autophagy). Finally, the upregulation of innate and adaptive immune responses were identified supporting the inflammatory environment in the aged retinae. The upregulation of MHC class II antigens, expressed only on the antigen-presenting cells (APCs), suggests the infiltration of immune cells in the aged retinae. Among the DEGs, upregulation of complement factors including C1s, C2, C3, and C4a, C4b, C1r, Cfh, Cfi, C1rl, was seen in the aged retinae (Supplementary Data File 2). Activation of the complement system is a significant factor contributing to age-related macular degeneration. In agreement with the immunostaining data, we observed upregulated expression of the markers activated microglia/macrophages, Cd68 and Aif1 (encoding IBA1), as well as the marker of activated Müllar glia, Gfap, in the aged retina (Supplementary Data File 2). The downregulated DEGs in the aged vs. young retinae affected BPs related to the retina or eye function and development, as well as those in response to cell stress and cell adhesion (Fig. 9B), suggesting a compromised retinal blood barrier and reduced retinal function.

8-AG treatment led to detecting 80 downregulated and 87 upregulated DEGs compared to the untreated control (Supplementary Data File 3). 8-AG reversed aging-related pathways linked to cell stress, such as ERK1 & ERK2 cascade and MAPK activity, and reduced pro-inflammatory signaling, evidenced by lower levels of interferon-gamma, NF-KappaB, and chemokine production (Fig. 9C). Consequentially, the activation of microglia, astrocytes, T cells, neutrophils, leukocytes, and B cells was downregulated by 8-AG, along with downregulation of phagocytosis and angiogenesis, showing a potent anti-inflammatory effect. Upregulated DEGs were involved in oxygen transport, neural processing, and anti-inflammatory pathways (Fig. 9D), with notable transcripts including growth hormone-releasing hormone receptor (Ghrhr) (47, 48), Gpr171 (49), and Mir-124 (50, 51), known for neuroprotective and anti-inflammatory properties (Supplementary Data File 3). Collectively, these changes suggest that 8-AG alleviates cell stress and exhibits a broad-spectrum anti-inflammatory effect and these effects could all contribute to retinal protection.

### The transcriptome of RPE/choroids suggests an age-related weakening of tight junctions and upregulated inflammatory signals, partially reversed by 8-AG.

**Figure 10:**
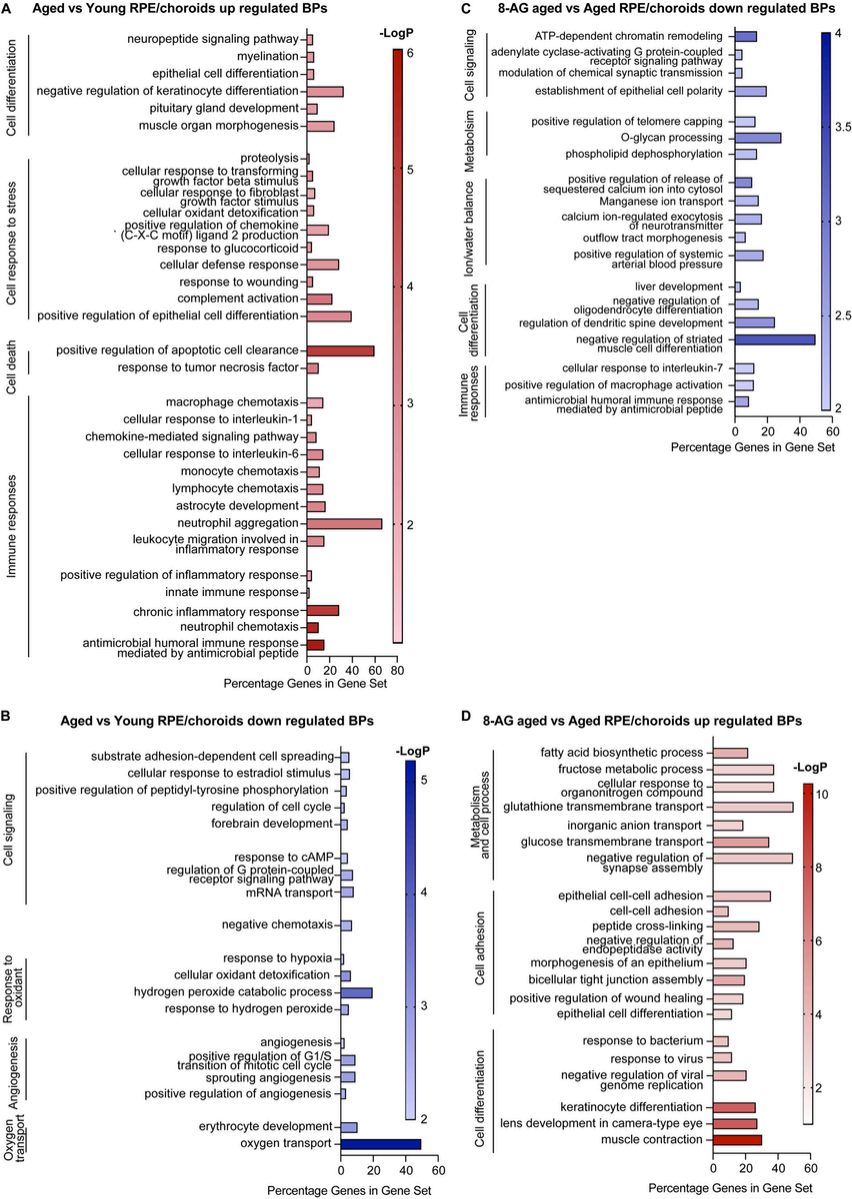
Transcriptomics of RPE/choroid reveals partial recovery of declined tight junctions and attenuation of upregulated inflammatory signals in 8-AG treated rats. RPE/choroids of young, aged, and 8-AG-treated aged rats were manually separated from the retinae were processed for transcriptomic analysis using bulk RNA-seq. Aged untreated vs young and aged 8-AG treated vs aged untreated DEGs were analyzed by GO to identify affected biological pathways (BPs). DEGs were identified with >1.5-fold changes with *P*-values <0.05. **A** and **B** are up- and down-regulated BPs, respectively, in RPEs/choroids from aged untreated vs young rats. **C** and **D.** are up and down-regulated BPs, respectively, in RPEs/choroids in aged 8-AG treated vs aged untreated rats. The X-axis represents the DEGs percentage in a gene set and the Y-axis represents the BPs. On the right side of all the graphs list the cellular pathways.

RPE/choroid tissue identity was confirmed by the enrichment of RPE-specific mRNAs, including Cst3, Efemp1, Itgav, Crispld1, Itgb8, Gulp1, Rpe65, Best1, Rbp1, Rlbp1, Rgr, Lrat, Pmel, Tttr, Tyr, Tyrp1, and Ptgds (Supplementary Data Files 4 and 5) (52). Comparing aged vs young rat RPE transcriptomes, we identified 166 upregulated and 118 downregulated DEGs (Supplementary Data File 4). Three known AMD-associated genes, C3, Cfi, and Mbp (52), were upregulated in the aged rat RPE/choroids. GO pathway analysis of the upregulated DEGs points to immune responses, including complement activation (C3, C4b, and Cfi), antimicrobial humoral immune response mediated by antimicrobial peptide (Camp, Ccl27, Cxcl1, Cxcl6, Reg3g, S100a9), response to tumor necrosis factor (Ccl2; Chi3l1; Mbp; Ubd), astrocyte development (Gfap; S100a8; S100a9), cellular response to transforming growth factor beta stimulus (Cdkn2b; Ovol2; Postn; Wnt10a; Wnt2), and so forth (Fig. 10A and Supplementary Data File 4). Aged downregulated DEGs suggested down-regulation of hydrogen peroxide catabolic process (Hba-a2; Hbb; Pxdn), angiogenesis (Angpt2; Aplnr; Col15a1; Enpep; Tek), negative chemotaxis (Flrt2; Itgb3; Sema3g), regulation of cell cycle (Ccnd2; Dusp1; Mecom; Plcb1; Skil; Xiap), cell adhesion mediated by integrin (Adam17; Itgb3), response to hypoxia (Adam17; Adipoq; Alas2; Angpt2; Loxl2; Tek), as shown in Fig. 10B and Supplementary Data File 4. These changes suggest decreased tight junction and decreased anti-inflammatory responses in the aged RPE/choroid.

8-AG treatment downregulated 140 genes and upregulated 519 genes in aged RPE relative to controls (Supplementary Data File 5). The downregulated DEGs suggest the down-regulation of pathways including cell signaling and metabolism, ion/water balance, cell differentiation, and immune responses. In contrast to the aging-associated upregulation of pathways involved in immune responses, 8-AG treatment led to downregulated immune responses including responses to IL-7, macrophage activation and antimicrobial humoral immune response (Fig. 10C). The 8-AG downregulated BPs include the ion homeostasis which potentially affects water balance and blood pressure (Fig. 10C), and 8-AG is known to modulate blood pressure and water-ion balance (53).. 8-AG upregulated genes were involved in metabolism, cell adhesion and differentiation, including genes encoding tight junction proteins like claudins (Cldn2, Cldn3, Cldn4, Cldn7, Cldn19 and Cldn23, Supplementary Data File 5). Cldn-19 is dominantly expressed in RPEs (54, 55). These changes suggest 8-AG may restore RPE and choroid capillary junction integrity, thereby improving the retina-blood barrier.

### Age-related accumulation of hypoxanthine and xanthine is accompanied by the age-related decline of guanine and 3’5’-cGMP.

**Figure 11:**
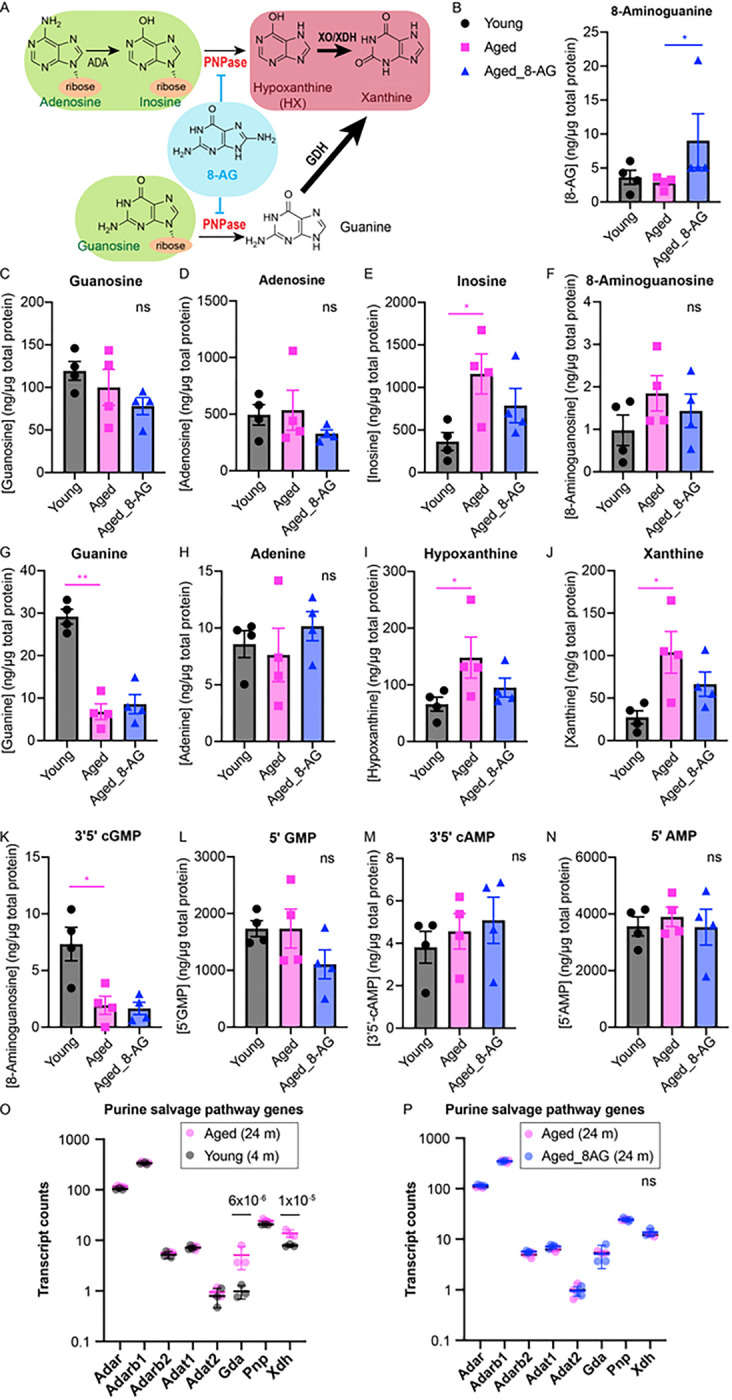
8-AG increases retinal levels of 8-AG (protective purine) and decreases levels of hypoxanthine and xanthine (damaging purines) in Fisher 344 rats. Retina from young, aged and 8-AG treated aged rats were harvested, lysed in buffer, and processed to assess the purine metabolome using UPLC-MS/MS. **A.** Cartoon shows the catabolism of purine nucleosides, inosine, and guanosine. The tissue-protecting purines are shown in green color background, tissue-damaging purines are in red color background, 8-aminoguanine (8-AG), in light blue color background shows the inhibition of PNPase in the reaction cascade. **B-N.** relative comparison of retinal purine metabolites from young (black circle), aged (magenta square) and aged rats treated with 8-AG for 8 weeks (blue triangle), normalized with the total protein in micrograms (ng of metabolite/μg of total protein). **B.** Quantity of 8-AG. **C.** Quantity of guanosine. **D.** Quantity of adenosine. **E.** Quantity of inosine. **F.** Quantity 8-aminoguanosine, **G.** Quantity of guanine. **H.** Quantity of adenine. **I.** Quantity of hypoxanthine. **J.** Quantity of xanthine. **K.** quantity of 3’5’-cGMP. **L.** Quantity of 5’-GMP. **M.** Quantity of 3’5’-cAMP. **N.** Quantity of 5’-AMP. N=4, statistical analysis to compare between samples was done using Kruskal–Wallis, *, *P*<0.05, ns is non-significant, data points and error bars are means and SDs, respectively. **O.** Bar plot of transcripts involved in the purine salvage pathway from young (black) and aged (magenta) rat retinae, using RNAseq data. **P.**Bar plot of transcripts involved in the purine salvage pathway from aged (magenta) and 8-AG treated aged (blue) rat retinae, using RNAseq data. N=3. Data and error bars are means+SD. Significant differences are labeled with *P*-values calculated by one-way ANOVA.

To assess the effects of aging and 8-AG treatment to the retinal purine metabolites, for the first time in record, we quantitatively profile the purine metabolome of the retina using UPLC-MS/MS (Fig. 11). We found the abundant purine metabolites in the rat retina are 5’-AMP, 5-’GMP, inosine and adenosine (Fig. 11D,E,L,N). Compared to the young retinae, the aged rat retinae showed a substantial reduction in guanine (> 3 fold), and 3’5’-cGMP levels (> 3 fold, Fig. 11G&K), with a rise in inosine (> 2 fold), hypoxanthine (~ 2 fold) and xanthine levels (~ 3 fold, Fig. 11E,I,J), whereas the levels of remaining purines were not affected significantly. As hypoxanthine is the product of inosine, the age-related accumulation of hypoxanthine is due to the age-related accumulation of inosine (Fig. 11A). Interestingly, the RNA-seq data showed that while genes encoding the adenosine deaminases (Adar, Adarb1, Adarb2, Adat1, Adat2), and the PNPase (Pnp) are not affected by aging, the expression of guanine deaminase (Gda) and xanthine dehydrogenase (Xdh/Xo) were 5-fold and 1.7 fold in the aged retinae, respectively, compared to the young retinae (Fig. 11O and Supplementary Data File 2). Higher expression of Gda can lead to higher consumption of guanine and a higher production of xanthine, while a higher level of Xdh can lead to a higher rate of production of xanthine from hypoxanthine. These results suggest age-related accumulation of hypoxanthine and xanthine is due to the age-related accumulation of inosine and elevated Gda and Xdh levels, which results in guanine drop and xanthine accumulation. 8-AG treatment increased its own retinal levels, suggesting the retinal bioavailability of 8-AG (Fig. 11B). 8-AG didn’t reverse the decline of guanine and 3’5’-cGMP in the aged rat retinae (Fig. 11G&K). Although 8-AG slightly reduced PNPase products, hypoxanthine, and xanthine, (Fig. 11I&J), it did not increase PNPase substrates inosine and guanosine (Fig. 11C&E), suggesting the retinal protection by 8-AG include other mechanisms in addition to direct PNPase inhibition within the retina, possibly including peripheral inhibition. Indeed, peripheral inhibition of PNPase, for example in the erythrocytes which are a rich source of PNPase, by 8-AG could be involved in the mechanism of action of 8-AG in the retina.

### Long-term efficacy of 8-AG in Fischer 344 rats.

**Figure 12:**
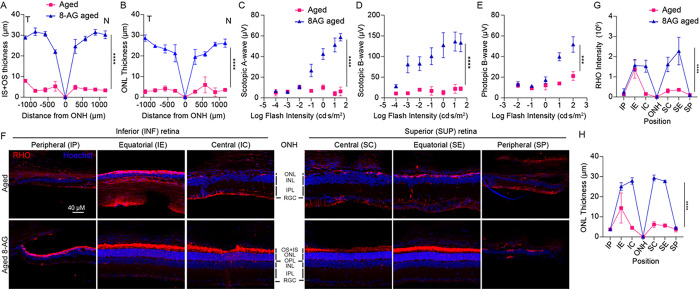
Long-term efficacy of 8-AG in Fischer 344 rats. The experimental Fischer 344 rats were untreated aged or aged treated with 8-AG at 5 mg/kg bw daily in drinking water for 17 weeks starting at 23 months of age. Rats were euthanized at the end of treatment (27 months) and SD-OCT, ERG, and IHC for rhodopsin (RHO) were performed. **A** and **B.** are the spidergrams for OS+IS thickness and ONL thickness, respectively measured from volumetric SD-OCT scanning. T and N represent temporal and nasal side of the retinae. magenta squares, aged rats; blue triangles, 8-AG treated aged rats. **C-E.** ERG response for scotopic a-wave **(C)**, scotopic b-wave **(D)**, and photopic b-wave **(E)**plotted as a function of flash intensity in semi-log format. **F.** Immunofluorescence images of RHO (red) and Hoechst 33342 for nucleus (blue) at central (SC&IC), equatorial (SE&IE) and peripheral (SP&IP) regions of the aged untreated and aged 8-AG treated rat retinae. **G** and **H.** Spidergrams of rhodopsin intensity in the OS+IS and ONL thickness **(I)**, measured from IHC images shown in **F.** Data points and error bars are means and SEMs. N=2 for untreated retinae, N=4 for 8-AG for treated retinae. ****, 0.0001 by two-way ANOVA between aged untreated and aged 8-AG treated. (This data set has a low sample size because the average rat life span is 24 months, only one-two animal survived at 27 months for each group).

Following the beneficial effects observed after 8 weeks of treatment in 24-month-old rats, we extended the study to assess if 8-AG maintains effectiveness through the rats’ lifespan. Specifically, we examined the F344 rats’ retinal structure and function after 17 weeks of treatment starting at 23 months (Fig. 12). Due to high mortality past 24 months, only one untreated (n = 2 eyes) and two treated (n = 4 eyes) rats survived for final analyses. Spectral domain-optic coherence tomography (SD-OCT) revealed maintained retinal structure in 8-AG-treated rats, while untreated ones showed significant degeneration (Fig. 12A&B). Scotopic and photopic ERG responses in 8-AG-treated rats indicated preserved rod and cone functions, contrasting with the near-complete loss of responses in untreated rats (Fig. 12C–E). IHC at 27 months showed abnormal localization of rhodopsin, loss of OS, and zero to one row of ONL nuclei in untreated rats, whereas 8-AG-treated retinae retained rhodopsin localization in the OS and 6–7 rows of ONL nuclei, despite no rescue in peripheral areas (Fig. 12F–H). Collectively, the long-term treatment with 8-AG showed even higher efficacy in preserving the structure and function of the aged retinae.

### 8-AG confers temporary retinal protection in the Rho^P23H/+^ knock-in mouse model of retinitis pigmentosa.

**Figure 13:**
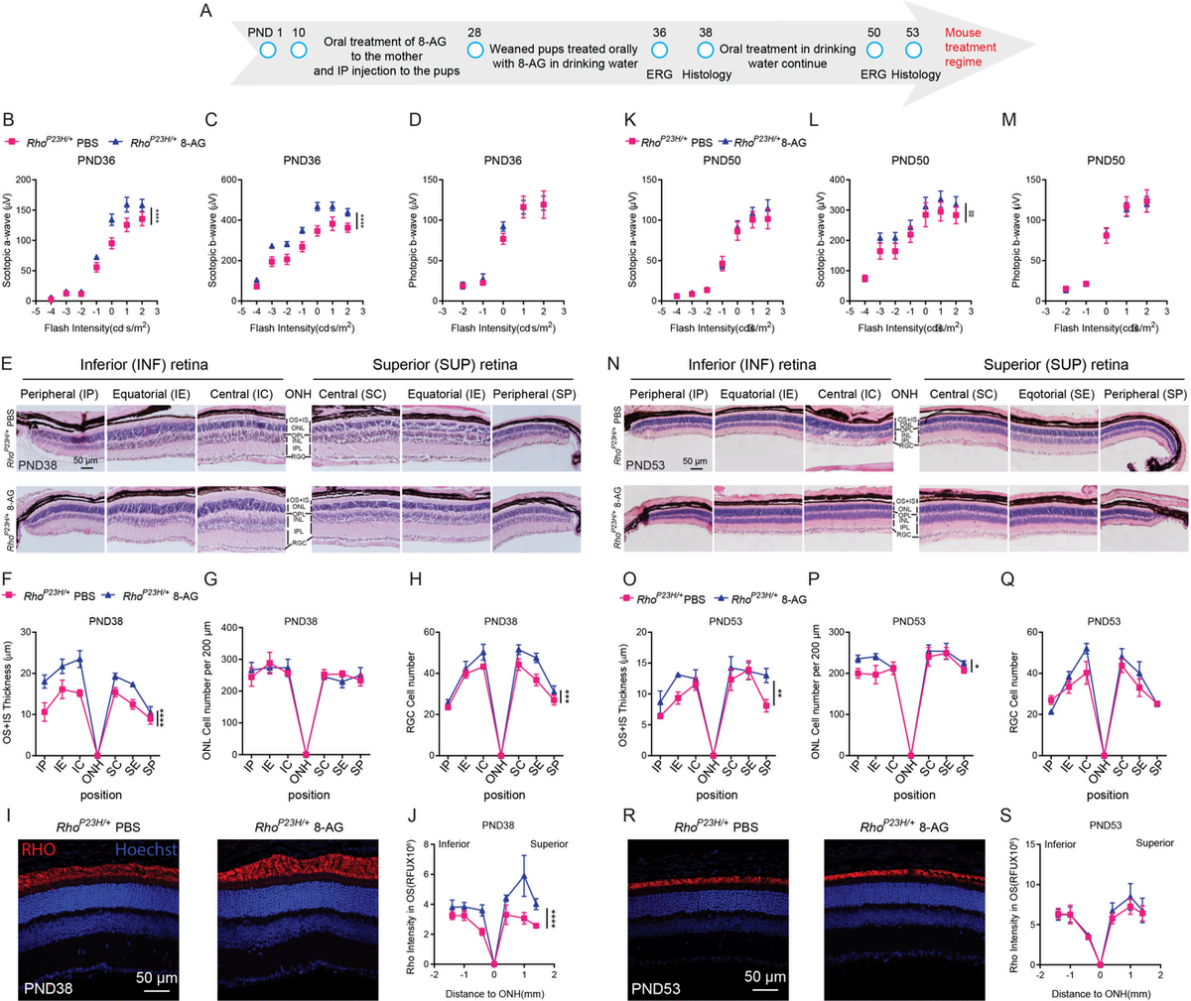
8-AG protects retinae of *Rho*^*P23H/+*^ knock-in mice. **A.** *Rho*^*P23H/+*^ mice treated with 8-AG at 11 mg/kg bw daily starting at PND 10 as shown in the treatment regimen in **A.** ERG recordings were performed at PND 36 and 50 for animals with 2 and 4 weeks of treatment, respectively. Mice were euthanized and eyes were enucleated at PND 38 and PND 53 for H&E staining or immunohistochemistry on retinal cross-sections. PBS-treated animals are used as vehicle control. **B-J** are data set obtained from animals treated with 8-AG or vehicle for 2 weeks. **K-S** are data sets from animals treated with 8-AG or vehicle for 4 weeks. **B**&**K, C**&**L,** and **D**&**M** are scotopic a-wave, b-wave and photopic b-wave responses plotted as a function of flash intensity in semi-log format at PND 36 and 50, respectively. Magenta squares, PBS-treated *Rho*^*P23H/+*^ mice; and blue triangles, 8-AG-treated *Rho*^*P23H/+*^ mice. N=4. **E**&**N** are hematoxylin and eosin staining images on the superior (S) and inferior (I) sides of optic nerve head (ONH) at central (SC&IC), equatorial (SE&IE) and peripheral (SP&IP) regions of retinal paraffin sections, Scale bar, 50 μm. **F**&**O, G**&**P,** and **H**&**Q** show the spidergrams of OS+IS thickness, ONL cell number, and RGC cell number per 200 μm retinal section, respectively, at PND 38 and 53. **I**&**R** are the representative immunofluorescence image of mice retinal cross-sections at central position at PND38 and 53, respectively. Red, RHO; and blue, Hoechst33343 for nucleus staining. Scale bar, 50 μm. **J**&**S** are spidergrams of RHO intensity in OS at different positions of the retinae generated from I and R, respectively. N=5. Data points and error bars are means and SEMs, respectively. *, **, ***, ****, P<0.05,0.01,0.001, and 0.0001, respectively, by two-way ANOVA.

We then tested 8-AG’s efficacy in the *Rho*^*P23H/+*^ knock-in mouse model of retinitis pigmentosa (RP), a different retinal degeneration model caused by rhodopsin misfolding (Fig. 13 and S16-S18) (56). Daily intraperitoneal (i.p) injections from PND10 to 28, followed by oral administration of 8-AG in drinking water, until PND38 or PND53. 8-AG treatment led to enhanced scotopic a- and b-waves at PND 36 (*P* < 0.0001), but only scotopic b-wave improvement persisted at PND 50 (Fig. 13B,C,K,L), suggesting a short- term rod function enhancement by 8-AG. No impact on photopic b-waves was seen, suggesting no effects of 8-AG on cone function in this model, similarly as observed in F344 rats (Fig. 13D&M). Retinal histology showed significantly increased OS + IS thickness at PND 38 and 53 (Figs. 13E,F,N,O, S16 and S17). The ONL cell count was slightly increased by 8-AG at PND 53, but not at PND 38 (Fig. 13E,G,N,P). Interestingly, 8-AG also led to a higher number of neurons in the RGC layer at PND 38 (*P* < 0.001) and PND53 (not significant, Fig. 13E,H,N,Q). Immunostaining showed that RHO level in the OS + IS increased with 8-AG treatment at PND 38 (Figs. 13I&J and S18, *P* < 0.0001), but not at PND 53 (Fig. 13R&S and S18). Interestingly, the time point when RHO level was increased by 8-AG can be directly correlated with the time when scotopic ERG responses were increased by the treatment (Fig. 13B&C). Collectively, 8-AG’s retinal protection appears limited to short-term, in the *Rho*^*P23H/+*^ knock-in mouse model of RP.

### 8-AG treatment reduces activated microglia/macrophages in Rho^P23H/+^ mice.

**Figure 14:**
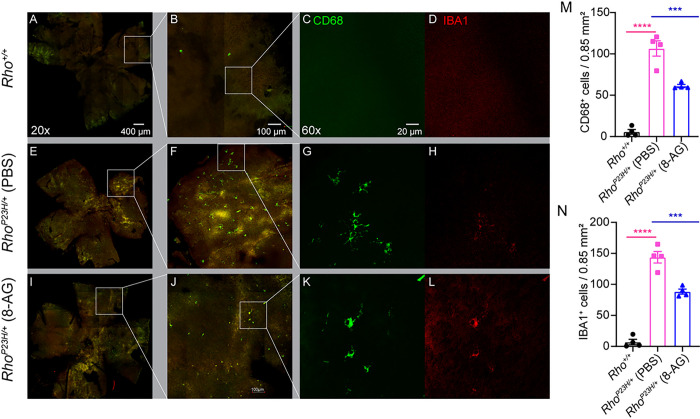
8-AG reduces macrophages in *Rho*^*P23H/+*^ knock-in mice. *Rho*^*P23H/+*^ mice were treated with 8-AG at 11 mg/kg bw via daily IP injections from PND 10 to 28 followed by oral 8-AG treatment (Fig 11A). Mice were euthanized and eyes were enucleated for retinal flat mount immunostaining. **A-L.** Immunofluorescence images of retinal flat mounts stained with CD68 and IBA1 as markers of microglia/macrophages. **A-D.** Top panels, retina from *Rho*^*+/+*^ mice; **E-H** middle panels, retina from *Rho*^*P23H/+*^ mice treated with PBS control; and **I-L** bottom panel, retinae from *Rho*^*P23H/+*^ treated with 8-AG. Images from left to right are stitched composite images of whole retinae (A, E, and I) from images obtained at 20x objective with the scale bar at 400 μm; enlarged retinal flat-mount images (B, F, and J) with composite channels of red and green under 20x objective with the scale bar at 100 μm; immunofluorescence of CD68 staining (**C, G,** and **K**) in green under 60x objective with the scale bar at 20 μm; and immunofluorescence images of IBA1 (**D, H,** and **L**) staining in red under 60 x objective. White squares indicate the regions of where the enlarged images shown on the right were taken. **M** and **N.** Bar graphs of CD68^+^ and IBA1^+^ cells number per 0.85 mm^2^ area, respectively. N=4. Bar heights and error bars are means and SDs, respectively. ***, *p*<0.001 and 0.0001, respectively, analyzed by one-way ANOVA.

We then asked whether 8-AG has any effects on the microglia/macrophages in this animal model (Figs. 14 and S19). Similar to our previous report (57), *Rho*^*P23H/+*^ mouse retinal flat mounts exhibited over 10-fold increase in CD68^+^ cells and IBA1^+^ cells compared to normal *Rho*^*+/+*^ mice, with IBA1^+^ cells outnumbering CD68^+^ cells (Figs. 12A–H,M,N and S19). As CD68 and IBA1 are markers of activated microglia/macrophages, this result suggests the activated microglia/macrophages increased 10-fold in the *Rho*^*P23H/+*^ mouse retinae. Treatment with 8-AG roughly halved the population of both cell types (Fig. 12E–N), mirroring the anti-inflammatory effects observed in F344 rat retinae and suggesting that 8-AG’s anti-inflammatory action may be effective across different models of retinal degeneration.

## Discussion

This study, extending from its established multi-tissue protective benefits, highlights the retinal protective effects of 8-AG. We showed strong morphological and functional retinal protection by 8-AG in the aged F344 rats. To be noted, the treatment was oral supplementation in the water, at a low dose (5 mg/kg/day), and the intervention was mainly tested at an old age (22–24 months). Eight weeks of oral treatment with 8-AG led to a significantly higher number of photoreceptor cells in the ONL, thicker OS/IS, more cones and cells in the RGC layer, enhanced scotopic ERG responses, and fewer apoptotic cells compared to controls. This marks the first identification of an orally administered small molecule with high potency and efficacy in preventing age-related retinal degeneration in an established natural model of aging. The reduction in MDA and 8-OHdG immunostaining indicates 8-AG’s antioxidant action, while the decrease in CD68^+^ and IBA1^+^ cells, alongside RNA-seq data, points to its anti-inflammatory impact. 8-AG also showed short-term protection in the *Rho*^*P23H/+*^ genetic mouse model of RP, reducing microglia/macrophage activation.

The F344 albino rat strain exhibits severe age-related retinal degeneration, likely exacerbated by chronic light damage, a phenomenon also observed in albino mice (58). Our study found increased lipid and DNA oxidation in the inner retina and inner segments (IS). The oxidative damage can be caused by the free radicals generated by light under the albino background. A possible reason that lipid oxidation spares the OS is because of the constant OS renewal (59). Rod nuclei’s densely packed and highly transparent heterochromatin may protect DNA from light-related oxidative damage, while IS’s mitochondrial DNA is more exposed and thus more susceptible to light damage (60). 8-AG treatment reduced markers of tissue peroxidation (Fig. 6) but did not rectify mitochondrial morphological abnormalities in aged rats (Fig. 8). While 8-AG doesn’t reverse all aging-related changes, it does lower tissue oxidation and improves retinal function.

Previously we established a UPLC-MS/MS method for measuring purines (61), and here for the first time we used this method to profile the purine metabolome in the retina. We found that 3’5’-cGMP levels dropped significantly in aged retinae—a three-fold decrease compared to young ones (Fig. 11K). Given that 3’5’-cGMP is a vital secondary messenger for phototransduction, its depletion could further impair aged retinal function. Further, 8-AG decreases the accumulation of hypoxanthine and xanthine in the aging rat retinae, compounds associated with tissue oxidative damage (Fig. 11I&J). 8-AG’s antioxidant action in the neural retina may result from reducing these tissue-damaging purines. Our previous research indicates that oral 8-AG at 5 mg/kg/day increases purine nucleoside phosphorylase (PNPase) substrates and reduces PNPase products in the urine of F344 rats, suggesting it effectively inhibits PNPase systemically (31). (Note that PNPase should not be confused with polynucleotide phosphorylase1 (PNPT1), an enzyme that is sometimes referred to as PNPase and is involved in mRNA degradation). However, 8-AG treatment does not appear to inhibit total PNPase activity in the retina, as retinal inosine and guanosine levels remain unchanged (Fig. 11C&E). This could relate to the variable Pnp expression across different retinal cell types. Published single cell-RNA seq data of the retina and brain provides more clues that suggest Pnp expression is high in astrocytes, Müller glia and microglia, but low in neurons (62). Pnp levels are also high in immune cells such as T cells, B cells, neutrophils and macrophages (63). Inhibiting PNPase in these cells may lead to the accumulation of DNA-derived deoxyguanosine, dGTP, and subsequent suppression of DNA synthesis and cell proliferation (64). However, the potential impact of 8-AG on inhibiting PNPase in these proliferative glial cells may not be apparent in overall retinal purine metabolome analyses due to their low abundance. Correlating to our RNA-seq data of retina and RPE/choroids pointing to 8-AG’s role in mitigating cell stress and inflammatory responses of immune cells, 8-AG’s anti-inflammatory effects may be due to its PNPase inhibition in the Müller glia, microglia, and infiltrated myeloid cells (T-cells, B-cells, and neutrophils) with high Pnp expression.

Chronic inflammation is a key hallmark of aging and is associated with increased susceptibility to many diseases (65). Chronic inflammation is involved in age-related degenerative diseases affecting “immune privileged tissue diseases” such as Alzheimer disease (66), Parkinson disease (67), as well as AMD (68). Although rodent models lack the macula, studying the impact of aging on retinal health offers insights relevant to AMD. Moreover, since aging is the primary risk factor for AMD, likely the effects of retinal aging in the rat model have some relevance to AMD. In this regard, aspects of our findings correlate to early AMD lesions including: 1) Upregulation of genes involved in complement factors (14) (C1qa, C1qb, C1qc, C1s, C1r, C2, C3, C3ar,C4a, C4b, Supplementary Data File 2); 2) activation of immune cells including microglia/macrophages (69) and lymphocytes (70) as evidenced by the increased number of CD68^+^ and IBA1^+^ cells in the neural retinal and choroid regions (Fig. 7C–F), as well as upregulation of genes involved in antigen processing and presentation of exogenous antigen via MHC class II (Fig. 9B); and 3) upregulated expression of AMD associated immunological genes (71) (Cfh, C2, C3, Cx3cr1, Tlr3 and Tlr4, Supplementary Data File 2). Notably, 8-AG treatment led to down-regulation of complement factors and their receptors (C1qa, C1qb, C1qc, C1qtnf7, C1rl, C3, C3ar1, C5ar1, C5ar2, Supplementary Data File 3), reduced the number of CD68^+^ and IBA1^+^ microglia/macrophages (Fig. 7C–F), down-regulated genes involved in the antigen processing and presentation of exogenous peptide antigen via MHC class II (Fig. 9D), yet not affecting all anti-inflammatory genes, such as Chf and Cx3cr1 (Supplementary Data File 3). Thus, it is likely that the anti-inflammatory effects of 8-AG are a major mechanism leading to 8-AG’s retinal protection in aged F344 rats.

In addition to its anti-inflammatory effects on immune cells, the bulk RNA-seq (Fig. 10) and TEM (Fig. 8) data also suggest that 8-AG regulates RPE cells by down-regulating RPE-mediated inflammatory signals, regulating retinal ion/fluid balance, upregulating tight junction genes as well as increasing phagocytosis flux. TEM images showed that 8-AG treatment reduced the number of undigested phagosomes in aged retinae, indicating improved phagocytosis. In RPE cells, these phagosomes often contain lipofuscin—a harmful aggregate of peroxidated photoproduct di-retinal A2E and oxidized lipids from ingested photoreceptor segments. Lipofuscin accumulation can trigger RPE cell apoptosis via NLRP3 inflammasome activation (72, 73), lysosomal proton pump inhibition (74) and lysosome alkalization (75), and is linked to retinal diseases such as Stargardt disease (76) and AMD (73). The RPE is adjacent to the choroid which has highly permeable choriocapillaris that could deliver 8-AG directly to the RPE. Inhibition of PNPase either in the the PNPase-enriched erythrocytes flowing through the choriocapillaris or in the RPEs (local diffusion of 8-AG into the epithelial layer) would increase inosine levels that can activate A_2_ receptors of RPE (31). The activation of A_2_ receptors has been reported to reacidify compromised lysosomes in the RPE (77) which can lead to increased phagocytosis flux. Future studies using A_2A_ and A_2B_ knockout rats or comparing the efficacy of 8-AG with the agonists and antagonists of A_2A_ and A_2B_ receptors will address this hypothesis.

Although we showed strong retinal protection by 8-AG in the natural rat model of aging, its potential in the pathological condition of AMD is still unknown. One important future study is to test 8-AG in an AMD model, such as the transgenic CFH-Y402H mouse (78), to evaluate 8-AG’s effects in AMD-related lesions such as sub-RPE deposit/drusen.

8-AG’s potential as a drug candidate was first discovered in the kidney, and then extended to diseases of the cardiovascular system and lower urinary tract (see (30, 79) for review). Here we show that 8-AG, an endogenous purine metabolite and an orally active drug candidate, reverses age-related retinal degeneration. The effects of 8-AG on the retina are likely mediated by multiple mechanisms including anti-oxidative, anti-apoptotic and anti-inflammatory effects. The present study indicates that age-related degeneration of an “immune privileged” tissue, i.e., the retina, can be mitigated via systems pharmacotherapy using 8-AG.

## Materials and Methods

### Animals.

All animal experiments followed the Animal Welfare Act and regulations guide and were approved by the University of Pittsburgh Institutional Animal Care and Use Committee (IACUC; rat protocol number 23073400, mouse protocol number 23053112). We obtained young and aged Fischer 344 rats from Charles River Laboratories and the National Institute on Aging [NIA]. Only female rats were used due to different timelines of retinal degeneration between females and males (40). The C57 black 6 (C57BL/6J) mice (*Rho*^*+/+*^, Stock no-000664) and (*Rho*^*P23H/P23H*^ knock-in, Stock no-017628) mice (56) were purchased from Jackson Laboratory. Both female and male mice were included in this study.

### Statistical Analysis.

Two-way analysis of variance (ANOVA) was applied to determine statistical difference between two groups of animals for the retinal thickness measured from OCT B-scans, the thickness of retinal layers, number of nuclei, and immunofluorescence intensity from retinal histology and IHC images, and ERG a- and b-waves. Two factors, treatment, and positions relative to optic nerve head (ONH, for OCT, IHC and retinal histology) were taken into consideration. Other data were analyzed by unpaired two-tail student’s t-test when comparing two groups of data, otherwise, Kruskal Wallis one-way ANOVA and multiple comparisons via the Dunn’s test were undertaken for three or more group of data. Data were presented as means ± SEMs or SDs as specified in the figure legends. Significant differences were determined when P value < 0.05 (*), 0.01 (**), 0.001 (***), or 0.0001 (****).

## Data Availability

RNAseq data generated in this study have been deposited in GEO and GEO accession number GSE254123. All procedures were approved by the University of Pittsburgh Institutional Biosafety Committee (IBC): IBC201700072. The rest of the materials and methods are described in Supplementary Materials.
